# Income-related inequalities in diseases and health conditions over the business cycle

**DOI:** 10.1186/s13561-017-0150-x

**Published:** 2017-03-09

**Authors:** Tinna Laufey Ásgeirsdóttir, Hildur Margrét Jóhannsdóttir

**Affiliations:** Department of Economics, University of Iceland, Oddi v/Sturlugotu, Reykjavík, Iceland

**Keywords:** Equality, Distribution, Health, Diseases, Income, Business cycles, I1, I14, I15, E3

## Abstract

How business cycles affect income-related distribution of diseases and health disorders is largely unknown. We examine how the prevalence of thirty diseases and health conditions is distributed across the income spectrum using survey data collected in Iceland in 2007, 2009 and 2012. Thus, we are able to take advantage of the unusually sharp changes in economic conditions in Iceland during the Great Recession initiated in 2008 and the partial recovery that had already taken place by 2012 to analyze how income-related health inequality changed across time periods that can be described as a boom, crisis and recovery. The concentration curve and the concentration index are calculated for each disease, both overall and by gender. In all cases, we find a considerable income-related health inequality favoring higher income individuals, with a slight increase over the study period. Between 2007 and 2009, our results indicate increased inequality for women but decreased inequality for men. Between 2009 and 2012 on the contrary, men’s inequality increases but women’s decreases. The overarching result is thus that the economic hardship of the crisis temporarily increased female income-related health inequality, but decreased that of men.

## Background

As the income gap between the richest and the poorest widens, the importance of monitoring health disparities across the income spectrum rises, and the debated amount of appropriate government spending on health care makes income-related health inequality an even more relevant subject to study. The importance of tracking how health is distributed along the income spectrum is made even clearer by the fact that the potential gains from publicly provided health care is not only improved public health but also minimized socio-economic health disparities.

Although studies on the income-related distribution of health in general have been extensive and the knowledge of the effects of business cycles on health has improved, the understanding of how business cycles affect income-related distribution of diseases and health disorders is much more limited. Using data from a survey on health and well-being conducted by the Directorate of Health in Iceland, this paper seeks to explain how the prevalence of thirty diseases is distributed across the income spectrum. Specifically, we examine this during a very turbulent economic period in Iceland, including a boom (2007), a subsequent bust (2009), and a period of partial recovery (2012). We thus take advantage of the unusually sharp change in economic conditions in Iceland during the Great Recession in 2008 and analyze how income-related health inequality changed across the different time periods.

As our study combines income inequality, health and business cycles, it can be seen as a contribution to at least two strands of literature; on the one hand, the relationship between income (income inequality) and health, and on the other hand, the relationship between business cycles and health. Therefore, the literature review is split in three parts; firstly, it discusses previous studies on the association between income inequality and health, secondly, it provides an insight into business cycles’ effects on health and lastly, income-related health inequality and business cycles will be discussed followed by a brief discussion on the Icelandic context.

### Health, income and inequality

A range of studies indicate a positive correlation between income and health, although the causality remains hard to determine [1–4]. Many economists refer to social factors such as education and labor-market participation as the most important determinants of this correlation and point out that educated individuals are better suited to understand health information and hence in a better position to reap the benefits of health-care systems and choose a healthy life style than their less educated counterparts [5, 6]. Those same individuals also tend to have higher income, and many argue that higher income discourages risky health behavior and reduces the risk of health disorders, although health behavior has also been associated with income independent of education [7–9]. The reason here can for example be that incentives may be relatively greater to prolong life that is lived in relatively better economic circumstances.

The abovementioned hypothesized pathways focus on individual-level determinants of income-related health disparities. However, those may be suppressed or elevated by society-wide determinants, such as social, cultural and institutional settings. Although not conclusive at this point, a body of literature has indicated that in general, worse health can be found in societies with more unequal income distributions and numerous studies have concluded that more equal societies not only have better health [3, 4, 10] but also less health inequalities [11, 12]. Wilkinson and Pickett [4] argue that the association between income inequality and health is strongest when income inequality is measured across whole societies and that better health in more equal societies can be explained through stronger social relationships, social cohesion, trust, lower violence rates and less social distance.

Ásgeirsdóttir and Ragnarsdóttir [13] examine concentration indices for 26 European countries and find relatively extensive income-related health inequality in the Nordic countries, a result that has also been found by other researchers [14–18]. Ásgeirsdóttir and Ragnarsdóttir [13], shed light on the relationship between societal conditions and income-related health inequality, for example GDP. However, they do this cross-sectionally across countries. Although this is relevant, especially as Iceland is one of those Nordic countries examined, the focus of the current project is on rapidly changing ambient economic conditions within a country. In that regard it should be noted that Ásgeirsdóttir and Ragnarsdóttir [13] did not find a systematic pattern between countries’ GDP and income-related health inequality. However, although GDP across countries may not be associated with the inequalities examined, this does not have to be the case for GDP levels over time within the same country – namely the business cycle. It may very well be that a variable that is generally positively associated with GDP across countries is counter-cyclical within a country. In fact health is a case in point, as population health generally increases with increased prosperity, while still being countercyclical by many estimates as discussed in the following section.

### Health and business cycles

A wide range of studies has shown that recessions generally have a positive impact on public health [1, 9, 19–27]. Ruhm and Cawley [9] add that in accordance with these findings, mortality rates have proven pro-cyclical, driven by mortality that can be explained by life-style decisions, such as coronary heart disease and traffic accidents. Ruhm and Gerdtham [27] studied macroeconomic fluctuations and health in the OECD countries and reported that decreases in unemployment rate during the period 1960–1997 are associated with substantial increases in death caused by diseases such as cardiovascular disease, liver disease, influenza as well as deadly accidents and Ruhm [26] shows that over the period 1972–1991, temporary economic downturns in the United States reduced eight of ten sources of fatalities.

Ruhm and Cawley [9] suggest two mechanisms likely to explain these counter-cyclical health effects; firstly, they mention that less disposable income during recessions reduces unhealthy consumption (see also [28, 29]), secondly, they argue that during recessions, people tend to have more time for healthy behavior (e.g. exercise), since working hours are usually pro-cyclical (see also [30–32]). Others have pointed out that health can be seen as a production input, and hence when working hours are extended, people’s health is affected, especially in cyclically sensitive sectors (such as construction), which indeed tend to be characterized by unhealthy or even dangerous working conditions [27, 33–35]. Ruhm [36] emphasizes that although the general results show that health improves in hard times, there are studies that indicate that mortality has become less pro-cyclical over the last few years (e. g. [37]) and even countercyclical [38, 39]. The important fact should also be mentioned that the results appear to be somewhat disease specific; Ruhm [26] shows that although physical health appears to be countercyclical, mental health has often been measured as pro-cyclical and Ruhm [36] finds that mortality due to cancer has over the last years become counter-cyclical, and suicide rates declined in economic upturns.

### Income-related health inequality and business cycles: The Icelandic context

Although the effect of business cycles on income-related health inequalities has received much less attention, Ásgeirsdóttir’s and Ragnarsdóttir’s recent study [40] does focus on income-related inequality in health in Iceland using data from 2007 to 2009, a year pre and post the Icelandic economic collapse. Their results indicate pro-rich income-related health inequality and they find that the effects of the crisis substantially differ between males and females. For men, income-related health inequality increased after the crisis while for women it remained rather stable. They use data from surveys conducted in 2007 and 2009, but as a first attempt at this examination, they only use a very general one-variable health measure, namely self-assessed health. They call for further research examining this relationship using more specific measures of health. Such an analysis is presented in the current study.

Iceland’s economy was hit hard by the Great Recession with considerable societal effects. Icelanders experienced, in a relatively short period of 5 years, an economic boom, followed by a sudden bust and then substantial recovery. These rapidly changing economic conditions make the years of 2007–2012 an ideal time period to examine the recession’s effects on income-related health inequality in Iceland. Although only Ásgeirsdóttir and Ragnarsdóttir [40] have examined the business-cycle effects of the collapse on income-related health distributions, the special research opportunity created by those dramatic circumstances in Iceland has been taken advantage of in multiple studies examining the effect of the crisis on health [13, 41–44] and health behaviors [45–50]. Due to the incredibly low pre-crisis unemployment rate (of only 2% in October 2007) the large percentage increase in unemployment during the crisis still resulted in a lower rate (of 6% in October 2009) than in most other hard-hit countries such as Spain and Ireland [46, 51]. However, while these countries experienced a deflation during the crisis, in Iceland, on the contrary, prices rose due to a devaluation of the Icelandic Krona by about 36% in exchange markets [46]. This was evident both from the consumer price index which increased by roughly 12% between 2007 and 2008 and again 12% between 2008 and 2009 and from more than 10% drop in real wages between 2007 and 2010 [52]. While this obviously reduced people’s purchasing power, it also had detrimental effects on Icelanders’ debt obligations, especially those in foreign currencies [47]. Already by 2010, the CPI increase reverted back to its pre-crisis level (with annual increases of 3–5% between 2010 and 2012) and real wages to their pre-boom level [46, 47, 52]. This rapid recovery, which indeed was lauded in international news stories, has mainly been explained by three important factors: capital controls, a flexible labor market and flexible exchange rates [46, 53, 54].

Using data from the same survey as Ásgeirsdóttir and Ragnarsdóttir [40], as well as an additional wave of data from 2012, this paper seeks to extend the contribution of their study in two ways. Firstly, by extending the study period to include economic recovery. Secondly, by extending the depth of the analysis from one self-assessed health variable, to examining thirty different diseases and health disorders. These are eye disease (e.g. cataracts or glaucoma), irritable bowel syndrome, chronic fatigue syndrome, cold/flu, alcoholism (or substance addiction), chronic anxiety, chronic depression, anxiety, serious worries, sleeping difficulties, other mental disorders, shortness of breath, debility, myalgia, back/shoulder pain, arm pain, leg pain, frequent headaches, toothache, abdominal pain, rheumatoid arthritis, osteoarthritis, fibromyalgia, chronic back syndrome, chronic throat disease, diabetes, serious headaches, urinary incontinence, thyroid disease and high blood pressure. We examine the distribution of those diseases and conditions over the time period of interest by calculating concentration indices for each disease with respect to households’ disposable income, both gender specific and overall and comparing the indices across years.

The additional data from 2012 strengthens the study in various ways. It allows us to determine whether or not the effects of the collapse in 2008 reversed with the recovering economy. This is important, as it sheds light on whether observed changes are likely to be due to the economic conditions or underlying time trends in health distributions. Also, a limitation of the first two waves is that no additional individuals were sampled, leading to an overall aging of the sample between waves. With the additional wave from 2012, new participants with the same age range as the first sample in 2007 were added, allowing us to examine to what extent the changes are driven by the aging of the sample. As indicated above, the contribution of this study is partly the detailed analysis across different health conditions and diseases, whereas Ásgeirsdóttir’s and Ragnarsdóttir’s [40] study only used one general measure of self-assessed health. It should also be mentioned that although the relation between some of those diseases and income has already been examined, the changes in the income-related distribution of those diseases has not been examined specifically across the business cycle.

## Data

The data stems from the survey “Health and well-being”, conducted by The Directorate of Health in Iceland in October 2007, 2009 and 2012. The survey includes variables on health, demographics and income. In October 2007, a stratified random sample of 9.711 Icelanders received the questionnaire with a net response rate of 60,8%. In 2009, every participant who had agreed to be contacted again received a questionnaire again. That year, the response rate was 69,3%, corresponding to 42,1% of the original sample participating in the first two waves of the survey. In 2012, a third wave of the survey was sent to 10,093 subjects, 3,659 respondents from the original sample and a new sample of 6,434 participants. The response rate of the new sample was 55,0%, resulting in 3,537 new answers and the response rate of the original sample was 88,7%, resulting in 3,246 individuals answering all three waves. Having eliminated observations with missing values on key variables, the final sample consisted of 9,963 observations on 6,446 individuals. For each regression, the final sample then depended on the number of missing values for the particular variables applied in the calculations.

We use thirty binary variables for health dummies, each representing a specific health disorder or disease mentioned above, taking the value 1 if an individual is suffering from the disease and 0 otherwise. The income variable is derived from the question: In what range do you estimate your household’s income over the last 12 months? Fourteen response options ranged from “below 900 thousand” (7.064 USD) and “above 18 million” (141.287 USD) and the responses were coded as the midpoint of each interval. As the last option “above 18 million” had no upper bound, responses in that range were coded as 20,5 million.

In order to make households’ income comparable between different household compositions, the OECD modified equivalence scale was applied. The equivalence scale addresses the concept of economies of scale within the household and hence the notion that a household’s needs increase with every additional member, but not proportionally. To take this into account, each member was assigned a value (0–1) indicating his/her needs for resources relative to the others’; in particular, the first adult was assigned the value 1, every additional adult (over the age of 18) was assigned the value 0,5 and children were assigned 0,3. Although the OECD modified equivalence scale sets the child-adult cut-off at the age of fourteen, we categorized all individuals under the age of eighteen as children, due to data limitations. Subsequently, to obtain the equivalent household income variable used throughout the research, each household’s income was divided by the sum of all its members’ values making the income variable comparable between different household compositions as a measure of the financial means of the individual at hand [55].

## Methods

The methodology was based on the concentration curve and the concentration index. The concentration curve is built on the idea of the Lorenz curve but instead of plotting the cumulative portion of the population against cumulative portion of total income, it plots the cumulative portion of a health variable against the cumulative portion of the sample, ranked by income [56–59]. In our case, in order to address the relationship between the distribution of particular diseases and the distribution of income, the x-axis represents the cumulated population, ranked by household income (poorest-richest) and the y-axis represents cumulated health (the absence of one of the thirty diseases of interest each time). Thus, a straight diagonal 45° line would represent perfect income-related health equality (line of perfect equality) where every income group has the same prevalence of a certain disease. A curve below the line of perfect equality indicates better health concentrated among those with higher income and a curve above the line of perfect equality indicates better health concentrated among those with lower income.

The concentration index, derived from the concentration curve, resembles the Gini coefficient and quantifies the health-income inequality graphed by the concentration curve. The concentration index is defined as twice the area between the concentration curve and the line of perfect equality. It ranges between − 1 and 1 where 0 indicates perfect equality and − 1 and 1 stand for perfect inequality, favoring low income groups (−1) and high income groups (1). Formally, the concentration index is calculated by the following formula:

$$ C I=1-2{\displaystyle {\int}_0^1 L(s) ds} $$

where *L* (*s*) is the cumulative distribution of health, as a function of cumulative income, s. This can be computed by using the following formula, with individual level data:

$$ C I=\frac{2}{ n\mu}{\displaystyle \sum_{i=1}^n}{y}_i{R}_i-1 $$

with *y*_*i*_ (*i* = 1,.…,*n*) as the health score of individual *i*, μ as the mean level of health and *R*_*i*_ as the relative rank by individual *i*’s income [12, 18, 40, 57, 58].

Since our health variables are binary, calculations will be built on the Wagstaff Concentration Index (WCI), which is a slightly varied version of the concentration index. As Wagstaff [60] suggests for binary health variables, we normalize the CI by dividing it through by the reciprocal of the mean of the variable in question (1-μ):

$$ W C I=\frac{2}{ n\mu \left(1-\mu \right)}{\displaystyle \sum_{i=1}^n}{y}_i{R}_i-1 $$

The WCIs are calculated for each health variable each year, 2007, 2009 and 2012, followed by an inter-temporal comparison of the indices. As both health and labor-market outcomes can vary by gender, all estimations are run separately for males and females, as well as for the full sample. Having computed the WCIs of the 3 years we also compute the WCIs of the new-entry sample of 2012 (Sample 2), which had the same age range as the first sample (Sample 1) had in 2007. A comparison of the two samples gives us an idea of whether the changes in the WCIs of Sample 1 are partly driven by the aging over the 5 year period.

The WCI measurement does not take into account how unavoidable factors, such as age and sex, play a role in the inequality. This can be addressed using decomposition analysis, allowing us to partition the indices into unavoidable and avoidable inequality. Specifically, by decomposing the WCI into its various determinants, we isolate the unavoidable factors and calculate standardized WCIs by subtracting the unavoidable inequality due to gender and age. For each WCI, the decomposition is conducted using the following linear regressor model:

$$ {y}_i = \propto + {\displaystyle \sum_k}{\beta}_k{x}_{k_i} + {\varepsilon}_i $$

where *y*_*i*_ stands for the health measure for individual *i*, ${x}_{k_i}$ is a determinant of health for regressor *k*, and ε_*i*_ is the error term. The determinants used in the regression are equivalized household income, marital status, population density and number of people in household. Given *y*_*i*_ and ${x}_{k_i}$ we can rearrange and write the WCI as:

$$ W C I = {\displaystyle \sum_k}\left(\frac{\beta_k{\overline{x}}_k}{\mu}\right) W C{I}_k + \frac{GWC{I}_{\varepsilon}}{\mu} $$

where μ is the mean of the health measure *y*, $\overline{x}$ is the mean of the health determinant *x*_*k*_, *WCI*_*k*_ is the WCI for *x*_*k*_ (the contribution from each determinant *k* to income-related health inequality) and the residual is *GWCI*_ε_, the generalized WCI for the error term. The residual thus contains income-related health inequality not explained by variations in determinant *x*_*k*_ across the income spectrum [12, 18, 40, 57].

The data were analyzed in Stata 13.0 software [61].

## Results

Tables 1, 2 and 3 show summary statistics for males, females, and full samples respectively. Calculations are based on those who answered all the waves (Sample 1), and the last column, 2012a, shows new-entrants in 2012 (Sample 2).

**Table 1:** Summary statistics for men

	2007			2009			2012			2012a^a^		
Health dummies^b^	Mean	SD	N	Mean	SD	N	Mean	SD	N	Mean	SD	N
Eye disease	0,0467	0,2111	857	0,0432	0,2035	879	0,0367	0,1882	871	0,0396	0,1952	959
Irritable bowel syndrome	0,0177	0,1318	849	0,0148	0,1208	879	0,0172	0,1300	874	0,0137	0,1162	951
Chronic fatigue syndrome	0,0722	0,2590	845	0,0772	0,2670	881	0,0625	0,2422	864	0,0916	0,2886	961
Cold or flu	0,0939	0,2918	863	0,0841	0,2777	892	0,0655	0,2476	870	0,0983	0,2979	946
Alcoholism	0,0199	0,1397	855	0,0192	0,1373	885	0,0239	0,1529	878	0,0391	0,1940	971
Chronic anxiety	0,0873	0,2824	848	0,0783	0,2688	881	0,0710	0,2570	873	0,0912	0,2880	965
Chronic depression	0,0434	0,2038	853	0,0407	0,1977	885	0,0368	0,1883	870	0,0465	0,2106	968
Anxiety	0,1474	0,3547	875	0,1620	0,3687	895	0,1813	0,3855	888	0,2182	0,4133	976
Serious worries	0,1824	0,3864	877	0,2134	0,4099	895	0,2097	0,4073	887	0,2510	0,4338	976
Sleeping difficulties	0,1899	0,3925	874	0,2147	0,4108	899	0,2120	0,4089	887	0,2441	0,4298	975
Other mental disorders	0,0215	0,1451	837	0,0170	0,1295	880	0,0161	0,1260	868	0,0187	0,1355	963
Shortness of breath	0,0548	0,2277	876	0,0713	0,2574	898	0,0772	0,2670	881	0,0781	0,2685	973
Debility	0,1479	0,3552	872	0,1737	0,3791	898	0,1859	0,3893	882	0,1711	0,3768	976
Myalgia	0,2345	0,4239	870	0,2220	0,4158	892	0,2568	0,4371	880	0,2916	0,4547	967
Back/shoulder pain	0,3836	0,4865	876	0,3621	0,4809	892	0,3957	0,4893	887	0,4413	0,4968	979
Arm pain	0,1707	0,3764	873	0,1629	0,3695	896	0,1808	0,3851	885	0,1988	0,3993	981
Leg pain	0,2176	0,4129	873	0,2310	0,4217	896	0,2537	0,4354	887	0,2838	0,4511	976
Frequent headaches	0,0905	0,2871	873	0,0936	0,2915	897	0,0857	0,2801	887	0,1160	0,3204	974
Toothache	0,0526	0,2233	875	0,0512	0,2205	899	0,0415	0,1996	891	0,0603	0,2382	978
Abdominal pain	0,1039	0,3053	876	0,1000	0,3002	900	0,1003	0,3006	887	0,1296	0,3361	972
Rheumatoid arthritis	0,0438	0,2049	844	0,0637	0,2444	879	0,0778	0,2680	861	0,0734	0,2610	926
Osteoarthritis	0,0985	0,2981	853	0,1110	0,3143	883	0,1154	0,3197	858	0,1138	0,3178	940
Fibromyalgia	0,0202	0,1408	841	0,0331	0,1789	877	0,0225	0,1483	845	0,0325	0,1775	922
Chronic back syndrome	0,1307	0,3373	857	0,1456	0,3529	879	0,1731	0,3785	861	0,2002	0,4004	934
Chronic throat disease	0,0200	0,1401	850	0,0239	0,1529	878	0,0266	0,1611	864	0,0267	0,1613	936
Diabetes	0,0340	0,1813	853	0,0385	0,1925	883	0,0628	0,2427	860	0,0611	0,2396	950
Serious headaches	0,0505	0,2192	851	0,0397	0,1954	881	0,0682	0,2522	880	0,0706	0,2563	963
Urinary incontinence	0,0607	0,2389	857	0,0646	0,2459	883	0,0997	0,2997	863	0,0908	0,2875	947
Thyroid disease	0,0141	0,1181	849	0,0216	0,1456	878	0,0275	0,1635	874	0,0249	0,1560	962
High blood pressure	0,1809	0,3851	857	0,2102	0,4077	880	0,2315	0,4220	864	0,2329	0,4229	953
Average	0,1011			0,1061			0,1138			0,1283		
**Equivalized hh income** ^c^	5385	3068	890	4512	2429	913	4555	2510	908	4467	2486	1001

^a^ 2012a shows Sample 2 (the new-entrants in 2012)

^b^ Health dummies = 1 if the disease is present

^c^ Equivalized hh income stands for equivalized household income where the OECD modified equivalence scale has been used to make income comparable between different household compositions

**Table 2:** Summary statistics for women

	2007			2009			2012			2012a^a^		
Health dummies^b^	Mean	SD	N	Mean	SD	N	Mean	SD	N	Mean	SD	N
Eye disease	0,0619	0,2412	1017	0,0531	0,2244	1054	0,0709	0,2568	1015	0,0474	0,2125	1140
Irritable bowel syndrome	0,0486	0,2152	1008	0,0612	0,2398	1046	0,0326	0,1776	1013	0,0469	0,2114	1131
Chronic fatigue syndrome	0,0663	0,2489	996	0,0973	0,2965	1048	0,1132	0,3170	1007	0,1427	0,3500	1135
Cold or flu	0,1278	0,3340	1033	0,0977	0,2970	1065	0,1049	0,3066	1020	0,0965	0,2954	1130
Alcoholism	0,0158	0,1246	1015	0,0104	0,1015	1057	0,0078	0,0882	1021	0,0141	0,1180	1133
Chronic anxiety	0,0985	0,2982	1015	0,1150	0,3192	1061	0,1179	0,3226	1018	0,1492	0,3564	1133
Chronic depression	0,0531	0,2244	1016	0,0604	0,2384	1059	0,0560	0,2300	1018	0,0756	0,2644	1138
Anxiety	0,2353	0,4244	1037	0,2630	0,4404	1061	0,2666	0,4424	1039	0,2973	0,4572	1164
Serious worries	0,2543	0,4357	1046	0,2696	0,4440	1072	0,2963	0,4568	1043	0,3251	0,4686	1169
Sleeping difficulties	0,2878	0,4529	1046	0,3096	0,4626	1069	0,3244	0,4684	1042	0,3547	0,4786	1170
Other mental disorders	0,0249	0,1560	1003	0,0314	0,1745	1051	0,0248	0,1556	1008	0,0498	0,2177	1124
Shortness of breath	0,1055	0,3073	1043	0,1052	0,3069	1065	0,1245	0,3303	1036	0,1301	0,3366	1168
Debility	0,2374	0,4257	1032	0,2908	0,4544	1059	0,3475	0,4764	1033	0,2925	0,4551	1152
Myalgia	0,4609	0,4987	1037	0,4459	0,4973	1063	0,4672	0,4992	1036	0,5098	0,5001	1171
Back/shoulder pain	0,5077	0,5002	1038	0,4948	0,5002	1061	0,5388	0,4987	1043	0,5575	0,4969	1173
Arm pain	0,2439	0,4297	1029	0,2453	0,4305	1064	0,2792	0,2792	1035	0,2737	0,4460	1162
Leg pain	0,3041	0,4603	1039	0,2992	0,4581	1063	0,3295	0,4703	1041	0,3239	0,4682	1164
Frequent headaches	0,2144	0,4106	1040	0,2008	0,4007	1066	0,2056	0,4043	1041	0,2537	0,4353	1163
Toothache	0,0499	0,2178	1043	0,0551	0,2284	1070	0,0539	0,2260	1038	0,0748	0,2632	1176
Abdominal pain	0,1704	0,3761	1039	0,1717	0,3773	1060	0,1792	0,3837	1038	0,2257	0,4182	1161
Rheumatoid arthritis	0,0820	0,2745	1000	0,1161	0,3205	1051	0,1162	0,3206	990	0,1057	0,3076	1116
Osteoarthritis	0,1815	0,3856	1014	0,2098	0,4074	1058	0,2314	0,4219	1007	0,1906	0,3930	1133
Fibromyalgia	0,0887	0,2845	992	0,1099	0,3130	1046	0,1429	0,3501	994	0,1317	0,3383	1116
Chronic back syndrome	0,2000	0,4002	1015	0,1907	0,3930	1054	0,2510	0,4338	1016	0,2376	0,4258	1132
Chronic throat disease	0,0318	0,1755	1007	0,0313	0,1742	1054	0,0419	0,2005	1002	0,0486	0,2151	1132
Diabetes	0,0247	0,1554	1011	0,0274	0,1634	1057	0,0386	0,1928	1010	0,0328	0,1781	1129
Serious headaches	0,1098	0,3128	1011	0,1067	0,3089	1059	0,1308	0,3373	1017	0,1764	0,3813	1134
Urinary incontinence	0,1708	0,3765	1019	0,1627	0,3693	1057	0,2261	0,4185	1026	0,1793	0,3837	1138
Thyroid disease	0,0663	0,2490	1010	0,0755	0,2644	1059	0,1068	0,3090	1021	0,0884	0,2841	1142
High blood pressure	0,1896	0,3922	1023	0,2054	0,4042	1066	0,2542	0,4356	1011	0,2181	0,4131	1151
Average	0,1571			0,1638			0,1827			0,1883		
**Equivalized hh income** ^**c**^	4867	2877	1070	3938	2313	1091	4062	2259	1074	4123	2306	1197

^a^ 2012a shows Sample 2 (the new-entrants in 2012)

^b^ Health dummies = 1 if the disease is present

^c^ Equivalized hh income stands for equivalized household income where the OECD modified equivalence scale has been used to make income comparable between different household compositions

**Table 3:** Summary statistics for both sexes

	2007			2009			2012			2012a^a^		
Health dummies^b^	Mean	SD	N	Mean	SD	N	Mean	SD	N	Mean	SD	N
Eye disease	0,0547	0,2275	1883	0,0483	0,2145	1945	0,0561	0,2301	1891	0,0437	0,2046	2103
Irritable bowel syndrome	0,0343	0,1820	1866	0,0398	0,1954	1937	0,0254	0,1573	1892	0,0316	0,1751	2086
Chronic fatigue syndrome	0,0697	0,2547	1851	0,0876	0,2828	1941	0,0901	0,2864	1876	0,1195	0,3244	2101
Cold or flu	0,1128	0,3164	1906	0,0909	0,2875	1970	0,0865	0,2812	1895	0,0971	0,2962	2080
Alcoholism	0,0176	0,1314	1880	0,0143	0,1189	1954	0,0152	0,1225	1904	0,0256	0,1580	2108
Chronic anxiety	0,0929	0,2904	1872	0,0983	0,2977	1954	0,0960	0,2947	1896	0,1223	0,3277	2102
Chronic depression	0,0484	0,2147	1879	0,0511	0,2203	1956	0,0470	0,2117	1893	0,0621	0,2414	2110
Anxiety	0,1944	0,3958	1924	0,2161	0,4117	1967	0,2271	0,4191	1933	0,2607	0,4391	2144
Serious worries	0,2207	0,4148	1935	0,2437	0,4294	1978	0,2558	0,4364	1935	0,2910	0,4543	2148
Sleeping difficulties	0,2439	0,4296	1931	0,2663	0,4421	1979	0,2730	0,4456	1934	0,3039	0,4600	2149
Other mental disorders	0,0233	0,1508	1849	0,0247	0,1553	1943	0,0207	0,1426	1880	0,0354	0,1848	2091
Shortness of breath	0,0834	0,2765	1931	0,0896	0,2857	1975	0,1025	0,3034	1922	0,1063	0,3083	2145
Debility	0,1968	0,3977	1916	0,2362	0,4248	1969	0,2729	0,4456	1920	0,2364	0,4250	2132
Myalgia	0,3575	0,4794	1919	0,3437	0,4751	1967	0,3708	0,4832	1920	0,4108	0,4921	2142
Back/shoulder pain	0,4512	0,4977	1926	0,4336	0,4957	1965	0,4737	0,4994	1936	0,5042	0,5001	2156
Arm pain	0,2111	0,4082	1914	0,2079	0,4059	1972	0,2338	0,4233	1925	0,2389	0,4265	2147
Leg pain	0,2656	0,4418	1924	0,2684	0,4432	1971	0,2954	0,4563	1933	0,3055	0,4607	2144
Frequent headaches	0,1584	0,3652	1925	0,1519	0,3590	1975	0,1499	0,3571	1934	0,1906	0,3928	2141
Toothache	0,0518	0,2217	1930	0,0530	0,2241	1981	0,0481	0,2140	1934	0,0681	0,2520	2158
Abdominal pain	0,1391	0,3461	1927	0,1390	0,3461	1971	0,1430	0,3502	1930	0,1816	0,3856	2137
Rheumatoid arthritis	0,0648	0,2462	1853	0,0917	0,2887	1941	0,0987	0,2983	1855	0,0909	0,2876	2046
Osteoarthritis	0,1438	0,3510	1877	0,1639	0,3703	1952	0,1786	0,3831	1870	0,1560	0,3629	2077
Fibromyalgia	0,0576	0,2330	1841	0,0745	0,2626	1934	0,0873	0,2824	1844	0,0867	0,2815	2041
Chronic back syndrome	0,1684	0,3744	1882	0,1712	0,3768	1945	0,2153	0,4111	1881	0,2208	0,4149	2070
Chronic throat disease	0,0263	0,1599	1866	0,0288	0,1673	1943	0,0348	0,1832	1870	0,0386	0,1927	2072
Diabetes	0,0288	0,1674	1873	0,0338	0,1807	1953	0,0506	0,2193	1876	0,0461	0,2097	2083
Serious headaches	0,0828	0,2757	1872	0,0763	0,2656	1952	0,1015	0,3021	1901	0,1280	0,3341	2102
Urinary incontinence	0,1203	0,3254	1887	0,1189	0,3237	1952	0,1684	0,3743	1894	0,1388	0,3458	2089
Thyroid disease	0,0428	0,2025	1869	0,0513	0,2207	1949	0,0705	0,2561	1900	0,0597	0,2371	2109
High blood pressure	0,1858	0,3891	1889	0,2088	0,4065	1959	0,2440	0,4296	1881	0,2253	0,4179	2108
Average	0,1316			0,1374			0,1511			0,1609		
**Equivalized hh income** ^**c**^	5090	2974	1972	4195	2380	2019	4286	2386	1908	4282	2396	2203

^a^ 2012a shows Sample 2 (the new-entrants in 2012)

^b^ Health dummies = 1 if the disease is present

^c^ Equivalized hh income stands for equivalized household income where the OECD modified equivalence scale has been used to make income comparable between different household compositions

For the overall Sample 1, the frequency of the diseases and conditions increase over the period. For all 3 years, (and both samples) the summary statistics indicate higher prevalence of the diseases for women than for men.

This increase in disease prevalence is especially noteworthy between 2009 and 2012, thus on average, health is pro-cyclical between 2007 and 2009 but counter-cyclical between 2009 and 2012. This highlights the importance of having three waves to distinguish the effects of the changing economic environment from time trends, as well as the new sample of 2012 to shed light on the effects of the aging of the panel. Men’s health is not affected by the economic collapse in a substantial way, the average frequency in the diseases rises by only 4, 9% between 2007 and 2009 and increases by 7, 3% between 2009 and 2012. Women’s health is more volatile, with the average frequency increasing by 4, 2% between 2007 and 2009 and 11,5% between 2009 and 2012. Quite surprisingly, Sample 2 does not seem to be healthier than Sample 1 in 2012, despite the age difference. Although the trend in the average frequency of diseases is pro-cyclical between the first two waves and counter-cyclical between the latter two, the trends differ between diseases; for example, alcoholism is pro-cyclical over the whole period and mental illness (chronic anxiety, chronic depression and other disorders) has a counter-cyclical trend over the whole period.

Although the summary statistics are useful in order to study the impact of the recession on the prevalence of the diseases, our main results are listed in Tables 4, 5 and 6. These show the unstandardized and standardized Wagstaff Concentration Indices for each of the diseases/disorders. Table 4 represents the WCIs for men, Table 5 for women and Table 6 for the total sample. Again, the first three columns of the tables represent the WCI of Sample 1 (those who replied to the survey all three times) and the fourth column shows the WCI of Sample 2 (those who only replied in 2012).

**Table 4:** Wagstaff Concentration Indices for men

	2007			2009			2012			2012a^a^		
Disease	WCI	St. WCI^b^	SE	WCI	St. WCI	SE	WCI	St. WCI	SE	WCI	St. WCI	SE
Eye disease	0,1100	0,0848	0,0035	0,2308	0,2296	0,0031**	0,1460	0,0970	0,0030	−0,0836	−0,0744	0,0028
Irritable bowel syndrome	0,0941	0,0710	0,0028	−0,0164	−0,0148	0,0023	0,1031	0,0806	0,0026	0,1064	−0,0240	0,0021
Chronic fatigue syndrome	0,1400	0,1405	0,0053*	0,1571	0,1534	0,0053*	0,3214	0,2976	0,0043***	0,3219	0,3775	0,0052***
Cold or flu	−0,0194	0,0026	0,0068	0,1625	0,1619	0,0060**	0,1882	0,2073	0,0056**	0,1439	0,1059	0,0061**
Alcoholism	0,0037	−0,0113	0,0025	0,2208	0,2200	0,0025	0,2637	0,2545	0,0026	0,1352	0,1327	0,0034
Chronic anxiety	0,1196	0,1170	0,0062	0,0414	0,0411	0,0057	0,2358	0,2295	0,0055***	0,2854	0,2815	0,0056***
Chronic depression	0,1975	0,2107	0,0044*	−0,0083	−0,0104	0,0037	0,2491	0,2455	0,0036**	0,4187	0,4242	0,0040***
Anxiety	0,1169	0,1287	0,0084**	0,0211	0,0185	0,0090	0,0287	0,0224	0,0093	0,1804	0,1691	0,0107***
Serious worries	0,1470	0,1544	0,0094***	0,0330	0,0307	0,0108	0,1479	0,1521	0,0108***	0,2289	0,2235	0,0116***
Sleeping difficulties	0,0359	0,0338	0,0096	0,0019	−0,0003	0,0098	0,0555	0,0412	0,0100	0,1217	0,1230	0,0105***
Other mental disorders	0,4514	0,4566	0,0030***	0,1147	0,1124	0,0027	0,4338	0,4058	0,0022**	0,3323	0,2680	0,0028**
Shortness of breath	0,1649	0,1639	0,0046**	0,1965	0,1908	0,0053*	0,0393	0,0287	0,0052	0,2241	0,2584	0,0052***
Debility	0,1845	0,1801	0,0082***	0,1115	0,1079	0,0082*	0,2546	0,2450	0,0085***	0,2195	0,2215	0,0076***
Myalgia	0,1025	0,0489	0,0109**	0,0265	0,0633	0,0109	0,0389	0,0736	0,0121	0,0367	0,0141	0,0128
Back/shoulder pain	0,0541	0,0696	0,0152	0,0101	0,0113	0,0147	0,0590	0,0711	0,0161	0,0650	0,0624	0,0165*
Arm pain	0,0958	0,0957	0,0085*	0,0888	0,0869	0,0080	0,0309	0,0427	0,0093	−0,0716	−0,0240	0,0088
Leg pain	0,0936	0,1028	0,0104**	0,1150	0,1131	0,0106**	0,1917	0,1938	0,0106***	0,0368	0,0621	0,0111
Frequent headaches	0,1314	0,1589	0,0064*	0,0033	0,0059	0,0067	0,2547	0,2501	0,0065***	0,1937	0,1480	0,0074***
Toothache	0,1623	0,1654	0,0049*	0,4457	0,4507	0,0053***	0,2318	0,2694	0,0047**	0,1072	0,0981	0,0054
Abdominal pain	0,1460	0,1723	0,0068**	0,0821	0,0820	0,0068	0,1527	0,1581	0,0067**	0,0890	0,0719	0,0074
Rheumatoid arthritis	0,2086	0,2071	0,0037**	0,1866	0,1821	0,0043*	0,1738	0,1454	0,0048**	0,1693	0,2408	0,0044**
Osteoarthritis	0,1753	0,1422	0,0055***	0,2129	0,2088	0,0053***	0,2311	0,2058	0,0055***	0,1205	0,1967	0,0050*
Fibromyalgia	0,3319	0,3133	0,0021**	0,3122	0,3086	0,0027**	0,3818	0,3794	0,0024***	0,1474	0,2060	0,0027
Chronic back syndrome	0,1485	0,1672	0,0075***	0,1379	0,1380	0,0074*	0,1934	0,2123	0,0087***	0,1121	0,1312	0,0085**
Chronic throat disease	0,0977	0,0996	0,0026	0,1448	0,1446	0,0025	0,1150	0,1500	0,0033	0,0290	−0,0083	0,0027
Diabetes	0,0012	−0,0457	0,0029	0,0140	0,0120	0,0029	0,0311	0,0245	0,0040	0,1350	0,1767	0,0038
Serious headaches	0,1120	0,1350	0,0048	0,0166	0,0160	0,0042	0,1029	0,1149	0,0060	0,0576	0,0459	0,0056
Urinary incontinence	0,2260	0,1768	0,0042***	0,2070	0,2047	0,0040***	0,0977	0,0686	0,0051	0,1568	0,1879	0,0043*
Thyroid disease	0,4220	0,4047	0,0020**	0,2709	0,2690	0,0022*	0,1218	0,1104	0,0028	0,1162	0,1167	0,0022
High blood pressure	−0,0811	−0,1006	0,0089*	0,0322	0,0296	0,0090	−0,0231	−0,0542	0,0093	0,0668	0,1115	0,0080
Average	0,1391	0,1349		0,1191	0,1189		0,1617	0,1574		0,1401	0,1442	

*p < 0,10, ** < 0,05, *** < 0,01

^a^ 2012a shows Sample 2 (the new-entrants in 2012)

^b^ St. WCI stands for Standardized Wagstaff Concentration Indices where unavoidable inequality has been subtracted

**Table 5:** Wagstaff Concentration Indices for women

	2007			2009			2012			2012a^a^		
Disease	WCI	St. WCI^b^	SE	WCI	St. WCI	SE	WCI	St. WCI	SE	WCI	St. WCI	SE
Eye disease	0,2389	0,1496	0,0038***	0,2601	0,2269	0,0031***	0,2620	0,1362	0,0041***	0,1141	0,1477	0,0031
Irritable bowel syndrome	0,0916	0,1128	0,0046	0,1449	0,1644	0,0049**	−0,0630	−0,1670	0,0033	0,0927	0,0727	0,0038
Chronic fatigue syndrome	0,2531	0,2363	0,0047***	0,2522	0,2507	0,0055***	0,1833	0,1840	0,0061***	0,2606	0,2706	0,0065***
Cold or flu	0,1003	0,1200	0,0069*	−0,0154	−0,0165	0,0062	0,0553	0,0216	0,0065	0,1028	0,1054	0,0059*
Alcoholism	−0,0713	−0,0729	0,0025	−0,0341	0,0356	0,0020	0,1940	0,1295	0,0017	0,2782	0,3541	0,0018
Chronic anxiety	0,2669	0,2874	0,0059***	0,3014	0,2969	0,0063***	0,2358	0,2966	0,0068***	0,3738	0,3232	0,0071***
Chronic depression	0,3160	0,3123	0,0042***	0,2466	0,2303	0,0046***	0,2766	0,2671	0,0026***	0,4741	0,4493	0,0046***
Anxiety	0,0775	0,0924	0,0102*	0,0318	0,0390	0,0108	0,0872	0,1256	0,0113**	0,2084	0,0349	0,0116***
Serious worries	0,0763	0,1137	0,0107**	0,1322	0,1514	0,0113***	0,0690	0,0950	0,0122*	0,2195	0,1855	0,0122***
Sleeping difficulties	0,0652	0,0547	0,0114*	0,0884	0,0831	0,0117**	0,0664	0,0751	0,0128*	0,0775	0,0573	0,0125***
Other mental disorders	0,1665	0,1513	0,0032	0,4848	0,5487	0,0035***	0,3178	0,3165	0,0033**	0,3793	0,3267	0,0041***
Shortness of breath	0,1611	0,1741	0,0059***	0,1311	0,1022	0,0058**	0,0753	0,1142	0,0064	0,2043	0,1656	0,0068***
Debility	0,1066	0,1332	0,0099**	0,1517	0,1514	0,0111***	0,1678	0,2200	0,0126***	0,0773	0,0498	0,0110*
Myalgia	−0,0210	−0,0007	0,0169	0,0668	0,0852	0,0164**	0,0189	0,0724	0,0176	−0,0011	−0,0372	0,0180
Back/shoulder pain	0,0247	0,0379	0,0179	0,0276	0,0390	0,0181	0,0370	0,0677	0,0193	0,0761	0,0528	0,0191**
Arm pain	0,0666	0,0612	0,0100	0,0227	0,0079	0,0098	0,0473	0,0617	0,0110	0,0410	0,0573	0,0098
Leg pain	0,0629	0,0528	0,0115*	0,0374	0,0324	0,0114	0,0706	0,0814	0,0121*	0,0973	0,0981	0,0116***
Frequent headaches	0,0495	0,0938	0,0098	0,0599	0,0840	0,0095	0,0147	0,0557	0,0100	0,1125	0,0843	0,0107***
Toothache	−0,0280	0,0138	0,0044	0,1387	0,1794	0,0046*	0,2997	0,3474	0,0046***	0,3100	0,3061	0,0054***
Abdominal pain	0,0292	0,0736	0,0086	0,1747	0,2143	0,0088***	−0,0127	0,0287	0,0087	0,1375	0,1020	0,0099***
Rheumatoid arthritis	0,1498	0,1115	0,0050***	0,1324	0,0892	0,0055**	0,2328	0,2165	0,0059***	0,0410	0,0962	0,0050
Osteoarthritis	0,2062	0,1668	0,0074***	0,1916	0,1433	0,0075***	0,1785	0,0939	0,0085***	−0,0236	0,0305	0,0066
Fibromyalgia	0,1602	0,1236	0,0054***	0,2446	0,2071	0,0056***	0,1648	0,1231	0,0068***	0,0934	0,0900	0,0060*
Chronic back syndrome	0,1691	0,1759	0,0086***	0,1213	0,1080	0,0080***	0,1836	0,1705	0,0099***	0,1297	0,1238	0,0090***
Chronic throat disease	0,1325	0,1819	0,0033	0,1909	0,1814	0,0029*	0,2395	0,2018	0,0036***	0,1538	0,1647	0,0037*
Diabetes	0,1458	0,1359	0,0024*	0,2517	0,2373	0,0025***	0,1578	0,2615	0,0031*	0,0944	0,0737	0,0024
Serious headaches	0,2498	0,2764	0,0066***	0,0794	0,1078	0,0066	0,0838	0,1885	0,0075	0,0952	0,0765	0,0086**
Urinary incontinence	0,1189	0,0958	0,0077**	0,1234	0,1081	0,0071***	0,1686	0,1302	0,0088***	0,1777	0,1721	0,0071***
Thyroid disease	−0,0039	−0,0216	0,0045	0,0925	0,0889	0,0045	0,1384	0,1936	0,0055**	−0,0201	−0,0159	0,0047
High blood pressure	0,1460	0,1014	0,0078***	0,1078	0,0720	0,0079***	0,1332	0,0081	0,0096***	0,0315	0,0791	0,0072
Average	0,1169	0,1182		0,1413	0,1416		0,1361	0,1372		0,1470	0,1366	

*p < 0,10, ** < 0,05, *** < 0,01

^a^ 2012a shows Sample 2 (the new-entrants in 2012)

^b^ St. WCI stands for Standardized Wagstaff Concentration Indices where unavoidable inequality has been subtracted

**Table 6:** Wagstaff Concentration Indices for both sexes

	2007			2009			2012			2012a^a^		
Disease	WCI	St. WCI^b^	SE	WCI	St. WCI	SE	WCI	St. WCI	SE	WCI	St. WCI	SE
Eye disease	0,1948	0,1377	0,0026***	0,2486	0,2339	0,0022***	0,2411	0,1484	0,0025***	0,0318	0,0504	0,0021
Irritable bowel syndrome	0,1238	0,0292	0,0027**	0,1617	0,1563	0,0027**	0,0128	−0,0172	0,0021	0,1216	0,0756	0,0022
Chronic fatigue syndrome	0,1940	0,1905	0,0035***	0,2187	0,1878	0,0038***	0,2620	0,0508	0,0037***	0,2651	0,2690	0,0042***
Cold or flu	0,0905	0,1080	0,0048**	0,0660	0,0649	0,0043	0,1224	0,1116	0,0043***	0,1217	0,1261	0,0042***
Alcoholism	−0,0116	−0,0499	0,0017	0,1214	0,2213	0,0016	0,2213	0,2303	0,0015	0,1421	0,1778	0,0019
Chronic anxiety	0,2074	0,2020	0,0042***	0,2052	0,1979	0,0042***	0,2579	0,2510	0,0044***	0,3461	0,2894	0,0045***
Chronic depression	0,2718	0,2558	0,0030***	0,1755	0,1560	0,0030***	0,2809	0,2623	0,0029***	0,4548	0,4283	0,0031***
Anxiety	0,1123	0,1006	0,0066***	0,0433	0,0190	0,0070	0,0867	0,0772	0,0073***	0,2020	0,1664	0,0078***
Serious worries	0,1192	0,1203	0,0071***	0,0982	0,0963	0,0078***	0,1212	0,1178	0,0081***	0,2294	0,1963	0,0084**
Sleeping difficulties	0,0673	0,0364	0,0075**	0,0679	0,0426	0,0076**	0,0779	0,0515	0,0081***	0,1068	0,0762	0,0082***
Other mental disorders	0,2786	0,2419	0,0022***	0,3731	0,4085	0,0022***	0,3841	0,3307	0,0020***	0,3773	0,2958	0,0025***
Shortness of breath	0,1905	0,1858	0,0038***	0,1748	0,1334	0,0039***	0,0751	0,0681	0,0042	0,2241	0,1876	0,0043***
Debility	0,1564	0,1427	0,0064***	0,1623	0,1169	0,0068***	0,2257	0,1969	0,0075***	0,1523	0,1152	0,0066***
Myalgia	0,0685	0,0489	0,0098**	0,0852	0,0633	0,0096***	0,0564	0,0736	0,0105	0,0389	−0,0098	0,0107
Back/shoulder pain	0,0559	0,0568	0,0117**	0,0388	0,0269	0,0114	0,0649	0,0789	0,0125**	0,0815	0,0576	0,0125***
Arm pain	0,0965	0,0728	0,0065***	0,0658	0,0292	0,0063**	0,0521	0,0535	0,0072*	−0,0025	0,0139	0,0066
Leg pain	0,0869	0,0718	0,0077***	0,0812	0,0574	0,0077***	0,1320	0,1247	0,0080***	0,0725	0,0840	0,0080***
Frequent headaches	0,1133	0,1177	0,0059***	0,0812	0,0769	0,0058**	0,1229	0,1080	0,0060***	0,1609	0,1086	0,0064***
Toothache	0,0716	0,0794	0,0033	0,2854	0,3236	0,0035***	0,2728	0,3016	0,0033***	0,2015	0,1907	0,0038***
Abdominal pain	0,0941	0,1076	0,0055**	0,1602	0,1556	0,0055***	0,0733	0,0608	0,0055*	0,1371	0,0976	0,0062***
Rheumatoid arthritis	0,1812	0,1330	0,0031***	0,1667	0,1086	0,0035***	0,2176	0,1657	0,0038***	0,1009	0,1478	0,0034**
Osteoarthritis	0,2072	0,1485	0,0046***	0,2124	0,1492	0,0045***	0,2101	0,1253	0,0050***	0,0461	0,0843	0,0041
Fibromyalgia	0,2224	0,1485	0,0030***	0,2944	0,2158	0,0031***	0,2288	0,1478	0,0036***	0,1361	0,1016	0,0033***
Chronic back syndrome	0,1751	0,1733	0,0057***	0,1384	0,1211	0,0055***	0,1931	0,2067	0,0066***	0,1309	0,1307	0,0062***
Chronic throat disease	0,1270	0,1231	0,0021	0,1692	0,1266	0,0020*	0,1870	0,2152	0,0024***	0,1328	0,1205	0,0023*
Diabetes	0,0650	0,0407	0,0019	0,1185	0,1124	0,0019*	0,0863	0,1123	0,0025	0,1050	0,1273	0,0022
Serious headaches	0,2270	0,2291	0,0041***	0,1083	0,0862	0,0039**	0,1122	0,1405	0,0048**	0,1081	0,0614	0,0051***
Urinary incontinence	0,1737	0,0945	0,0044***	0,1849	0,1307	0,0041***	0,1746	0,1043	0,0051***	0,1881	0,1601	0,0041***
Thyroid disease	0,0908	0,0178	0,0025	0,1673	0,1103	0,0026***	0,1646	0,1599	0,0031***	0,0347	0,0096	0,0026
High blood pressure	0,0284	0,0060	0,0059	0,0624	0,0556	0,0059**	0,0642	−0,0069	0,0067**	0,0486	0,0821	0,0054
Average	0,1360	0,1123		0,1512	0,1328		0,1594	0,1350		0,1499	0,1341	

*p < 0,10, ** < 0,05, *** < 0,01

^a^ 2012a shows Sample 2 (the new-entrants in 2012)

^b^ St. WCI stands for Standardized Wagstaff Concentration Indices where unavoidable inequality has been subtracted

**Fig. 1:**
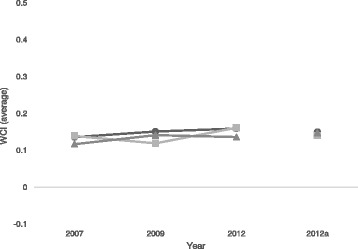
Average concentration indices

On average, for both genders, better health is concentrated among those with higher income over the whole period. Our results show an increase in income-related health inequality among women over the years 2007–2009, but a decrease (to a lesser extent than the increase) between 2009 and 2012. For men, on the contrary, inequality increased over the first period but decreased over the second, less than it rose over the first. On the whole, income-related inequality increased for both men and women from 2007 to 2012, as the increase exceeded the decrease for both gender, and therefore the overall indices suggest a steady increase over the whole period, first driven by the increase in women’s inequality and then by men’s. Figure 1 graphs the main results by showing the average WCIs, both by gender and overall.

**Fig. 2:**
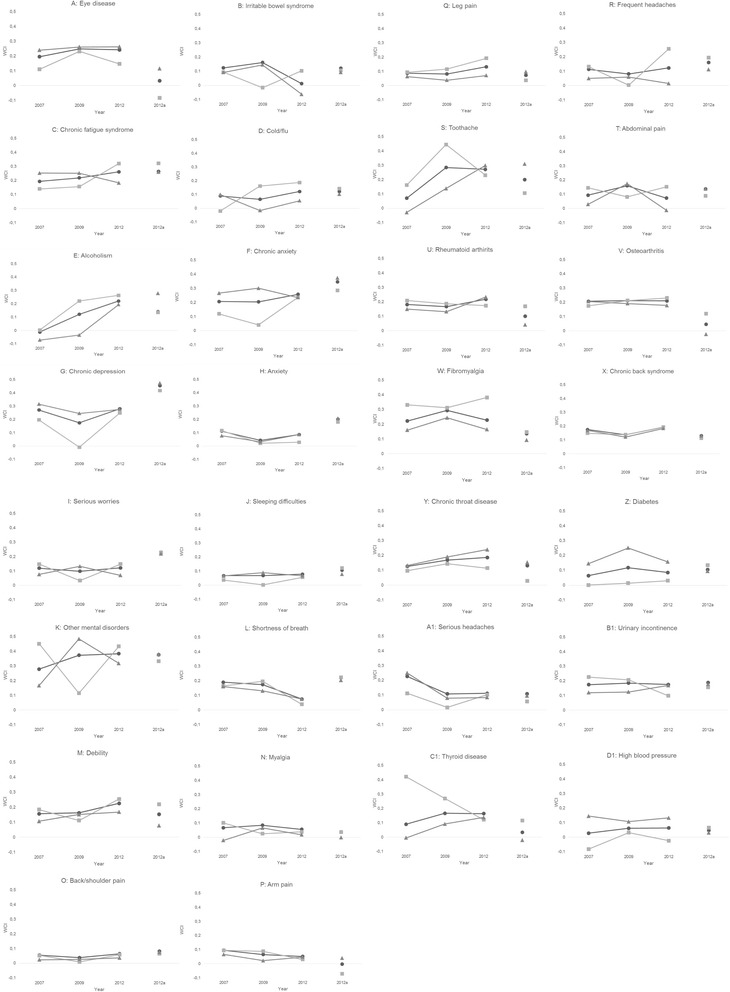
Diseases-specific concentration indices

The panels in Fig. 2 graph the trends of the disease specific WCIs, separately for males and females, as well as overall. With only few insignificant exceptions, they all show income-related inequality favoring higher income groups (with WCIs ranging from around zero to 0,5).

Relatively high income-related inequality can be seen for the mental conditions - chronic anxiety (panel f), chronic depression (panel g), and other mental disorders (panel k) - and in all cases the inequality is higher for women than for men. For alcoholism or substance addiction (panel e) the WCIs for both genders have increased steadily over the entire period and are substantially higher for males than females. Toothache (panel s) and fibromyalgia (panel w) have also relatively high WCIs for both genders, and again higher for men than women. The income-related inequality in toothache has a clear trend with a rapid increase around the recession, but a slight recovery between 2009 and 2012.

The most stable WCIs are those connected to physical pain - such as myalgia (panel n), back/shoulder pain (panel o), arm pain (panel p), leg pain (panel q), and both headache variables (panels r and a1) - and these WCIs tend to have a slight decrease between 2007 and 2009 and a small increase between 2009 and 2012. A relatively steady WCIs can be found for arthritis, both rheumatoid arthritis (panel u) and osteoarthritis (panel v).

## Discussions

Our findings contribute to existing studies on income-related inequality in health, and our research is – to our best knowledge – the first to look at the impact of an economic collapse on the income-related inequality in specific diseases and disorders. As mentioned earlier, the current study can be seen as an extension of Ásgeirsdóttir’s and Ragnarsdóttir’s paper [40] on the impact of the economic collapse on income-related health inequality in Iceland over the period of 2007–2009. With additional data from 2012 we were able to study the impact over a longer period, and while they used a general health measure we measure health specifically for thirty different diseases.

A large proportion of government spending in Western countries goes to health care, which makes it important to monitor socio-economic inequality, in particular income-related health inequality. As our study provides a valuable insight into the distribution of various diseases along the income spectrum in Iceland we hope our results can be of practical value.

The unusually sharp changes in the economic conditions in Iceland over the short time period examined allowed us to study the business cycle’s effects without being considerably affected by long-term changes in other variables which have caused methodological challenges in studies conducted over a longer time period. Furthermore, the timing of the collection of the data, which includes both income and health variables, enables us to take full advantage of these rapid changes as the first wave is collected a year before the economic collapse, the second wave a year after the collapse and the third 4 years after the crash, when the economy had already partly recovered.

Our results suggest a considerable income-related health inequality in Iceland over the years of 2007–2012. On average the higher-income individuals are healthier than their lower-income counterparts. On the whole, income-related health inequality increased steadily over the whole period, although the economic hardship of the crisis caused a temporary decrease in the case of males but an increase for women. For both genders, these temporal trends in the WCIs over the first period are reverted in the latter period during the recovery. It should also be mentioned that these opposing trends in the gender specific WCIs are most distinct in the trends of the mental health variables.

The gender differences in the WCIs highlight the importance of taking into account unavoidable factors by calculating standardized WCIs. For the full samples (both sexes), the standardized indices are substantially lower than the unstandardized ones. However, the differences between the standardized indices and the unstandardized are very small for the gender specific WCIs (where age is the only unavoidable factor). Clearly, the overall differences between the standardized WCIs and the unstandardized WCIs are driven by the large contribution of gender differences. In many cases, clear gender differences can also be observed in the impact of the recession on health, and our summary statistics reveal worse health and lower income for women than men over the whole period (results also found by Ásgeirsdóttir and Ragnarsdóttir [40]).

Furthermore, there are obvious gender differences in the trends of the WCIs; as mentioned above. During the economic downturn in the 2007–2009 period, women’s income-related health inequality increased while that of men’s decreased. During the recovery, in the 2009–2012 period, female inequality decreased and men’s increased. Although we can only hypothesize about the reasons behind the gender differences in the WCI trends, the decrease in men’s WCIs between 2007 and 2009 might be due to increased unemployment in cyclically sensitive sectors, such as construction industry, in which many studies have found counter-cyclical health trends as mentioned above. Increased unemployment in these sectors, which tend to be dominated by men, would then cause individuals to drop in the income ranking while improving in health, which lowers the WCIs. Our summary statistics support this hypothesis; physical health (especially physical health variables that are likely to be related to hard work such as myalgia, back/shoulder pain, arm pain, abdominal pain and serious headaches) improves during the recession (between 2007 and 2009). Regarding the increase in female income-related health inequality over the crisis period one might speculate whether these trends can be explained by different effects on different income groups: we suggest that women in higher income groups are more likely to follow Ruhm’s and Cawley’s [9] counter-cyclical health mechanisms mentioned above; during a recession they reduce their unhealthy consumption and increase healthy behaviors, such as exercise. Their lower income counterparts, on the other hand, might be more likely to be adversely affected by the recession, in particular less disposable income might affect their mental health. This hypothesis is also somewhat supported in our summary statistics; many of our physical health variables suggest that women’s physical health improves during the crisis but their mental health clearly deteriorates. The lower average income of females, relative to men, could potentially explain why a decrease in income during the crisis had larger effects on women’s mental health than men’s. However, the reason for this gender difference remains unclear and would be an interesting avenue for future research. Importantly, the fact that the average income-related health inequality for both genders reverts back towards their pre-crisis levels as the economy recovers from the recession (over the 2009–2012 period) makes the observed changes even more likely to be due to the economic conditions.

Quite surprisingly, the changes in our gender specific WCIs over the first period (2007–2009) are not in accordance with those of Ásgeirsdóttir and Ragnarsdóttir [40] who concluded a decreased inequality for women, but an increased inequality for men. Here it should be noted that it is in our WCIs for mental health (such as chronic anxiety (panel f in Fig. 2), serious worries (panel i in Fig. 2), other mental disorders (panel k in Fig. 2) and sleeping difficulties (panel j in Fig. 2)) that drive the rise in women’s WCIs and the decrease in men’s is most distinct. In this respect it is important to bear in mind that Ásgeirsdóttir and Ragnarsdóttir [40] use a very general self-assessed health measurement and people’s assessment is more likely to reflect physical health than mental health. This might partly explain the inconsistency between the results and highlights the importance of a more detailed analysis as the one presented here.

Our summary statistics show some consistency with the results of several studies mentioned earlier on the impact of business cycles on health; counter-cyclical physical health but pro-cyclical mental health [26, 27, 36]. In our results, such effects can be observed to some extent over the period 2009–2012. As Iceland’s economy recovered rapidly from the recession, physical health deteriorated, but some of our mental health variables indicate an improvement. However, over the first period, the average frequency of diseases remains very stable. The negative impact of the crisis on mental health is consistent with studies on other hard hit European countries such as Greece [62] and Spain [63]. However, a recent study [64] shows that in these countries, the crisis had larger adverse effects on health in general than in Iceland and suggests that this is due to the stark differences in fiscal policies adopted during the recession.

When crudely observing cyclicality from the summary statistics, the aging of the sample needs to be kept in mind. Despite the lower age range, Sample 2 has slightly higher frequency in diseases on average than Sample 1 in 2012 which gives a reason to assume that aging is not an important determinant in the health deterioration. Still, the diseases most likely to be correlated with aging (both kinds of arthritis, fibromyalgia, debility, urinary incontinence, thyroid disease, high blood pressure and eye disease) have lower frequencies in Sample 2. Interestingly, higher frequency of diseases in Sample 2 is evident in all of the mental illness variables.

It is important to note that our study employs self-reported measures of the diseases and conditions of interest, which, although widely used, might pose problems to the reliability of the measurements. Differences in health assessment might e.g. be affected by differences in people’s health awareness and evaluation, particularly since many of the health variables in question are conditions, rather than diseases. Moreover, one could even speculate whether such differences might be related to socio-economic status, which would further bias our measurements. Some conditions that have been of interest in previous research on business cycles and health are unfortunately omitted in this research, for example suicides, traffic accidents and infant mortality. How business cycles affect the distribution of those conditions across socioeconomic groups would however be of interest and we encourage future research on those topics.

The aging of the sample over the 5 year period can also be mentioned as a potential drawback to our study as the increase in the income-related inequality in health may partly be driven by the aging. One obvious reason for this is that as people grow older they are more likely to have worse health, and when they retire they tend to drop in their income ranking, which affects the income-related health inequality [65]. However, aging has also been found to increase the inequality before retirement. Deaton and Paxson [66] studied approximately 50.000 adults between the ages of twenty and seventy over the period of 1983 to 1994. They used self-reported health status as the health measurement and found that a correlation between health and income increases with age, particularly from the age of twenty up until retirement age. They provide several possible explanations for this; they mention how earnings are adversely affected by negative health shocks and hence progressively correlated with age. As people’s health after retirement does not affect income, they suggest that the correlation may weaken in very old age. They also evaluate possible causalities running from income to health such as how poorer people become more exposed to various risk factors with age and have more limited access to health care than their richer counterparts. As Sample 2 constitutes a group of participants in the same age range as that of Sample 1 in 2007, the presentation of this newest sample can be seen as our way to partly address this problem. Although not evident for females on average, Sample 2 has a slightly lower WCIs than Sample 1 does in 2012, which might give a reason to conjecture that the aging of the sample increases the income-related health inequality.

Our results suggest that the increase driven by the aging of the sample is more likely to be found in physical illness than mental illness, and especially evident in both kinds of arthritis, fibromyalgia and physical pain. Our standardized WCIs support these findings as they show that the contribution of age is stronger for men than women and much larger in the latter period than the first. Especially, the contribution of age increases the WCIs of Sample 1 in 2012. Thus, the increase in men’s income-related health inequality over the latter period is exaggerated by the aging of the sample. Consequently, as the overall increase in the income-related health inequality between 2009 and 2012 is driven by the biased increase in men’s inequality, it also needs to be discounted.

## Conclusions

By analyzing the period between 2007 and 2012 in Iceland, we show that the economic collapse increased female income-related health inequality but decreased that of men. These changes, which are largely driven by the trends in mental health inequality, reversed during the economic recovery period. Overall, we find a considerable income-related health inequality which increased steadily over the whole study period. Our results conform and contribute to a range of other studies on income-related inequality in health; they provide evidence of the effects of an economic collapse and a recovery on the distribution of thirty different diseases along the income spectrum. As many of the impacts and trends in the WCIs are still unexplained, a decomposition of all the WCIs would be a logical next step in order to provide a clearer picture of the main determinants of the income-related health inequality. Given the range of diseases and conditions examined within this study, such an analysis is beyond the scope of this paper, but encouraged.
